# RETRACTION: Roles of *Moringa oleifera* Leaf Extract in Improving the Impact of High Dietary Intake of Monosodium Glutamate-Induced Liver Toxicity, Oxidative Stress, Genotoxicity, DNA Damage, and PCNA Alterations in Male Rats

**DOI:** 10.1155/omcl/9854702

**Published:** 2025-11-06

**Authors:** Oxidative Medicine and Cellular Longevity

RETRACTION: T. Albrahim and M. A. Binobead, “Roles of *Moringa oleifera* Leaf Extract in Improving the Impact of High Dietary Intake of Monosodium Glutamate-Induced Liver Toxicity, Oxidative Stress, Genotoxicity, DNA Damage, and PCNA Alterations in Male Rats,” *Oxidative Medicine and Cellular Longevity* 2018, no. 1 (2018): 1–11. https://doi.org/10.1155/2018/4501097.

The above article, published online on 17 December 2018 in Wiley Online Library (wileyonlinelibrary.com), has been retracted by agreement between the journal's Chief Editor Jeannette Vasquez Vivar; and John Wiley & Sons Ltd. A third party reported that images of comet assays in Figure 5 showed evidence of manipulation. A different third party also reported further evidence that the comet assays were not possible in the actual experimental conditions. The editors agree that the comet assays show hallmarks of manipulation. The authors did not respond to an inquiry and request for original data by the publisher.

The retraction has been agreed to because the evidence of image manipulation and fabrication fundamentally compromises the editors' confidence in the results presented. The authors did not respond to the notice regarding the retraction.

